# Use of amantadine as substrate for SSAT-1 activity as a reliable clinical diagnostic assay for breast and lung cancer

**DOI:** 10.4155/fsoa-2018-0106

**Published:** 2018-12-11

**Authors:** Andrew W Maksymiuk, Paramjit S Tappia, Daniel S Sitar, Parveen S Akhtar, Nazrina Khatun, Rahnuma Parveen, Rashiduzzaman Ahmed, Rashid Bux Ahmed, Brian Cheng, Gina Huang, Horacio Bach, Brett Hiebert, Bram Ramjiawan

**Affiliations:** 1 Cancer Care Manitoba, Winnipeg, MB, R3E 0V9, Canada; 2 Department of Internal Medicine, Rady Faculty of Health Sciences, University of Manitoba, Winnipeg, MB, R3A 1R9, Canada; 3 Clinical Research Institute & Office of Clinical Research, St Boniface Hospital, Winnipeg, MB, R2H 2A6, Canada; 4 Department of Pharmacology & Therapeutics, Rady Faculty of Health Sciences, University of Manitoba, Winnipeg, MB, R3E 0T5, Canada; 5 Department of Medical Oncology, National Institute of Cancer Research & Hospital, Mohakhali, Dhaka, Bangladesh; 6 FastBios, Dhaka, Bangladesh; 7 BioMark Diagnostics Inc., Richmond, BC, V6X 2W8, Canada; 8 Division of Infectious Diseases, Faculty of Medicine, University of British Columbia, Vancouver, BC, V5Z 3J5, Canada; 9 Cardiac Sciences Program, Asper Clinical Research Institute, Winnipeg, MB, R2H 2A6, Canada

**Keywords:** amantadine, biomarkers, breast cancer, cancer diagnostics and screening, lung cancer, polyamine metabolism, SSAT-1

## Abstract

**Aim:**

Spermidine/spermine N^1^-acetyltransferase (SSAT-1) plays a critical role in cell growth, proliferation and death, and is known to be activated in human cancer cells. Amantadine, a US FDA-approved antiviral drug, is a substrate for SSAT-1 and can be used to indirectly measure SSAT-1 activity because of its conversion to acetylamantadine (AA). This study was undertaken to further validate SSAT-1 activity in breast and lung cancer patients.

**Results:**

An increase in the urinary concentration of AA in lung and breast cancer patients was observed. The 0–2 h collection time point was determined to be optimal in revealing significant differences in urinary AA concentration between healthy controls and cancer patients.

**Conclusion:**

The high urine concentration of AA could be used as a simple and useful test for the detection of breast and lung cancer.

## Lay abstract

This study describes a novel noninvasive urine test for detecting and screening of breast and lung cancer using a safe and approved drug called amantadine. Higher concentration of the acetylated form of amantadine in the urine are detectable in the urine of both breast and lung cancer patients as compared with healthy adult volunteers. This test is simple and may serve as a useful tool for determining the presence of breast and lung cancer.

## Graphical abstract

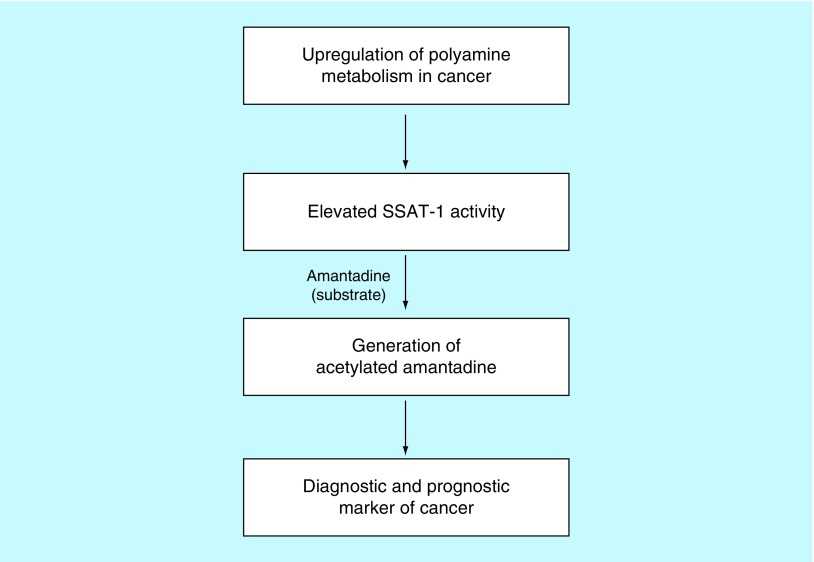

The American Cancer Society has reported that one in seven deaths globally can be attributed to cancer [1]. After excluding nonmelanoma skin cancer, the International Agency for Research on Cancer has estimated that there were 14.1 million new cases of cancer in the world in 2012, of which 8 million occurred in developed nations. Furthermore, deaths due to cancer in 2012 were 8.2 million (about 22,000/day). By 2030, the global burden of cancer is expected to be 21.7 million new cases and 13 million cancer deaths. Lung cancer is by far the foremost cause of cancer death among both men and women, accounting for 25% of cancer deaths. More people die of lung cancer than of colon, breast and prostate cancers combined each year [1,2]. Currently lung cancer ranks 13^th^ as a cause of death globally and, by 2040, is projected to rise to the 9^th^ leading cause of death [3]. On the other hand, breast cancer is the most common cancer in women worldwide, with nearly 1.7 million new cases diagnosed in 2012, making it the second most common cancer overall [4]. For women in the USA in 2017, a total of 252,710 new cases of invasive breast cancer were estimated, along with 63,410 new cases of breast carcinoma *in situ* and 40,610 breast cancer deaths per year [5].

Cost and access are important considerations in the implementation of screening for cancer in the general population. Conversely, the availability of an inexpensive, accurate, reproducible and simple test would be seen to be clinically and economically viable. Low-dose CT scans have been shown to be effective in reducing the mortality of patients at risk for lung cancer [6,7]. Despite this, general uptake and utilization of CT scans in screening has been disappointing, partly because of the cost, perceived risk of exposure to ionizing radiation, and false positives [7]. Also, there have been conflicting results obtained in various CT screening trials. In established screening programs in the US, the uptake by persons at risk of lung cancer is less than 50% [7]. This justifies the development of a ‘prescreen’ that could provide incentive and improve the specificity of CT screening.

Spermidine/spermine N^1^-acetyltransferase-1 (SSAT-1) is involved in the homeostasis of the polycationic aliphatic amines, spermine and spermidine. These polyamines are intimately involved in cell growth, proliferation and cell death [8,9]. Also, the upregulation of SSAT-1 in different types of cancer cells is well documented [10–12]. We have developed a urine test based on the catalytic activity of SSAT-1 to measure the increasing activity of the enzyme as evaluated by measurement of the acetylated products in urine. Thus, we hypothesize that its activity measurement can predict the presence of cancer and possibly the advancement of the disease. The abovementioned test is based on oral administration of a safe and single dose of a US FDA-approved drug amantadine. Amantadine is a specific substrate of SSAT-1, which is acetylated by the enzyme producing acetylamantadine (AA) [13], which is a terminal and stable end product excreted in urine [13,14].

Recently, we have reported the clinical utility of amantadine to detect elevated SSAT-1 activity by measuring increased concentration of AA in the urine of cancer patients. This increase was supported by the increase of the *SSAT-1* gene transcripts and protein contents in patient-derived tumor tissue [15]. While high levels of SSAT-1 gene and protein expression were measured in human primary breast, prostate and lung tumor tissue, an increase in the urinary concentration of AA in cancer patients was observed [15]. The present study was undertaken to optimize urine collection time points for AA and further validate the use of amantadine as a mean to detect increases in SSAT-1 activity. Thus, the determination of urinary AA concentration can be developed as a clinical test for detecting the presence of lung and breast cancer in humans.

## Materials & methods

### Regulatory & institutional review board approvals

Ethics approval was obtained from the University of Manitoba Biomedical Research Ethics Board (Ethics File #: B2012:063) prior to study implementation. The study protocol was reviewed and approved by Health Canada (File # 9427-B2749-21C); Notice of Authorization was dated 17 July 2012 and was also listed on the NIH Clinicaltrials.gov website (Identifier: NCT02277938). Appropriate Institutional Review Board (Ministry of Health & Family Welfare, the People's Republic of Bangladesh) approvals were also obtained (Ethics File # NICRH/Ethics/2017/288). Clinical studies were completed under Good Clinical Practice (GCP) and Good Laboratory Practice (GLP) conditions in accordance with the standards established by the Canadian Tri-Council Policies.

### Experimental subjects

Patients with newly diagnosed and untreated cancer were recruited into the study. At the Bangladesh site, 80 patients with lung cancer and 39 patients with breast cancer were recruited from the Department of Medical Oncology, National Institute of Cancer Research & Hospital, Mohakhali, Dhaka, Bangladesh. Healthy controls (n = 29) were recruited from within the local area. A total of 24 patients with lung cancer were recruited from CancerCare Manitoba (Winnipeg, Canada) and 20 healthy adult controls were recruited locally. All participants provided a signed informed consent for participation. Volunteers aged between 18 and 80 years were included in the study. Exclusion criteria were declared as follows: alcohol consumption within 5 days of amantadine ingestion; previous adverse reaction to amantadine; currently pregnant or lactating; and liver or kidney disease. On the day of the study, overnight-fasted participants were requested to provide a complete urine collection, prior to ingesting amantadine and then requested to orally ingest 200 mg (2 × 100 mg) amantadine capsules (mylan–amantadine, amantadine hydrochloride, USP). Complete urine was again collected at 2 and 4 h postamantadine ingestion for AA analysis. A 6 h postamantadine time point for urine collection was also included, but only at the Bangladesh site.

### Analytical procedures

Urine was analyzed for AA by established and validated GLP-compliant HPLC–MS methods using d_3_-AA as the internal standard (IS) for quantitation at Biopharmaceuticals Research, Inc. (Vancouver, BC, Canada; Study #: BIM-2015-001). Health Canada-authorized Biomark AA assay standard under application no: 229838 on 7 Oct 2014 (Investigational Testing Authorization). During the development of the LC–MS/MS assay for the quantitation of AA and amantadine in human urine, calibration standards were prepared over the concentration range from 0.1 to 100 ng/ml for AA and 0.08 to 24 μg/ml for amantadine plus blank controls, based on a volume of 1 ml human urine. Quality control samples in human urine were prepared at 0.4, 4, 20 and 80 ng/ml for AA and 0.32, 3.2 and 16 μg/ml concentrations for amantadine. All calibration standards, quality control samples and test samples were spiked with the IS, *N*-acetyl-d_3_-amantadine and processed by liquid–liquid extraction. Samples were analyzed using HPLC on a Synergi Hydro-RP 80Å 4 μm (50 × 2.0 mm, id column (Phenomenex, CA, USA), with tandem MS/MS detection using an electrospray ionization triple-quadruple mass analyzer (Agilent 1100, Agilent Technologies, CA, USA). Positively charged matrix factor (MF) and IS ions were monitored using the multiple reaction monitoring (MRM) mode. Quantitation of *ex vivo*-spiked AA and amantadine in human urine was performed based on the peak area response ratio of AA or amantadine to the IS added to all samples.

Total calibration variability was between -3.5 and 1.0% bias and not more than 6.4% CV from 0.2 to 100 ng/ml. At low level of quantification (LLOQ) level (0.1 ng/ml), a 4.0% bias and 11.0% CV was observed. Total assay accuracy and precision was between -1.0 and 12.0% bias and not more than 21.5% CV. AA recoveries from urine were satisfactory (84.4 to 87.8%) and reproducible (not more than [NMT] 2.2% CV).  Mass spectrometric detection of AA did not suffer from matrix effects (MF0.9220 to 1.0186, NMT 3.7% CV). Analyte recoveries from urine were stable in urine for 96 h at room temperature from and after up to three cycles of freeze/thaw in a range of pHs between 3 and 8 at 80 ng/ml. The LC–MS/MS assay method was successfully implemented for the measurement of AA in human urine collected from the present clinical study.

### Data cross validation

Cross validation of findings was performed with HPLC of urine samples for the presence of AA [16,17]. Samples were coded by the study staff and the technician analyzing the biological samples was blinded to any patient information other than the code on the label.

### Statistical analysis

**Figure 1.**
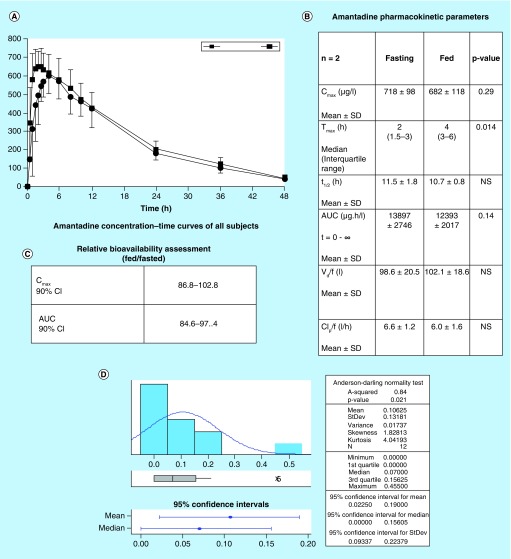
**Amantadine pharmacokinetics.** Amantadine pharmacokinetic parameters in normal healthy adults in fed or fasted state. Urinary acetylamantadine concentrations were determined by LC–MS/MS technique up to 12 h postamantadine ingestion. Values are mean ± SEM and are expressed as ng/ml. The p < 0.05 was considered as significantly different fed versus fasted state. AUC: Area under the curve; CL_p_/f: Apparent total clearance of the drug from plasma after oral administration; C_max_: Maximum (or peak) serum concentration that a drug achieves; t_1/2_: Time required for a quantity to reduce to half its initial value; T_max_: Time after administration of a drug when the maximum plasma concentration is reached; V_d_/f: Apparent volume of distribution.

**Figure 2.**
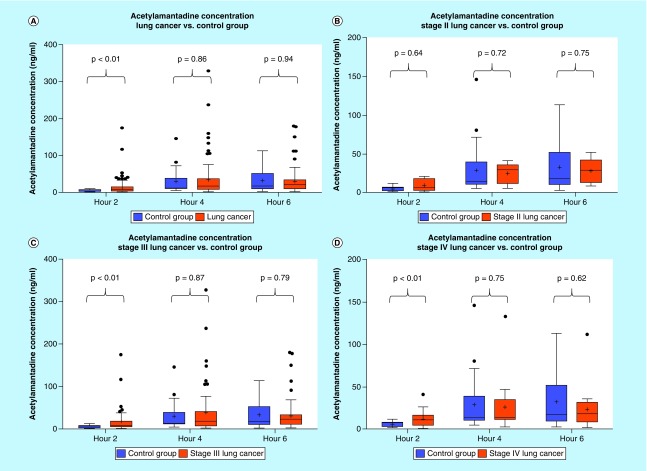
**Urinary acetylamantadine concentrations in patients with lung cancer from the National Institute of Cancer Research & Hospital (Dhaka, Bangladesh site).** Acetylamantadine concentrations are shown for all types lung cancer and staging **(A)**; Stage II **(B)**; Stage III **(C)**; and Stage IV **(D)**. Urinary acetylamantadine concentrations were determined by LC–MS/MS technique at 2, 4 and 6 h postamantadine ingestion. Values are mean ± SEM and are expressed as ng/ml. The p < 0.05 was considered as significantly different versus healthy control group. LC–MS/MS: Liquid chromatography–mass spectrometry/mass spectrometry;SEM: Scanning electron microscope.

**Figure 3.**
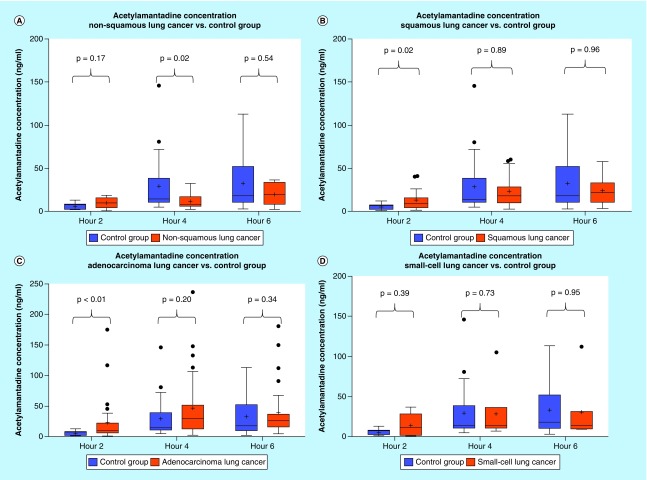
**Urinary acetylamantadine concentrations in patients with different subtypes of lung cancer from the National Institute of Cancer Research & Hospital (Dhaka, Bangladesh site).** Acetylamantadine concentrations are shown for nonsquamous lung cancer **(A)**; squamous lung cancer **(B)**; adenocarcinoma **(C)**; and small-cell lung cancer **(D)**. Urinary acetylamantadine concentrations were determined by LC–MS/MS technique at 2, 4 and 6 h postamantadine ingestion. Values are mean ± SEM and are expressed as ng/ml. The p < 0.05 was considered as significantly different versus healthy control group. LC–MS/MS: Liquid chromatography–mass spectrometry/mass spectrometry; SEM: Scanning electron microscope.

**Figure 4.**
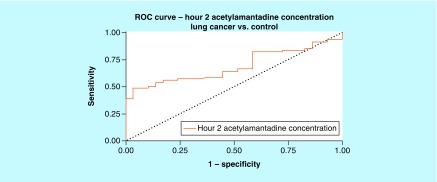
**Receiver-operating characteristic for acetylamantadine concentration at 2 h time point in lung cancer.** Area under ROC curve (95% CI) was demonstrated to be 0.689 (0.591–0.786). ROC: Receiver-operating characteristic.

**Figure 5.**
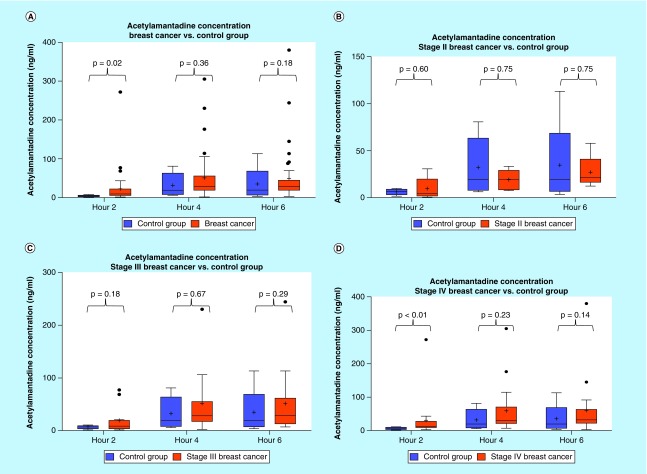
**Urinary acetylamantadine concentrations in patients with breast cancer from the National Institute of Cancer Research & Hospital (Dhaka, Bangladesh site).** Acetylamantadine concentrations are shown for all breast cancer cases **(A)**; and at Stage II **(B)**; Stage III **(C)**; and Stage IV **(D)**. Urinary acetylamantadine concentrations were determined by LC–MS/MS technique at 2, 4 and 6 h postamantadine ingestion. Values are mean ± SEM and are expressed as ng/ml. The p < 0.05 was considered as significantly different versus healthy control group. SEM: Scanning electron microscope.

**Figure 6.**
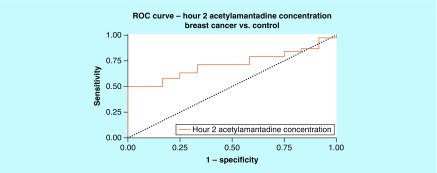
**Receiver-operating characteristic for acetylamantadine concentration at 2 h time point in breast cancer.** Area under ROC curve (95% CI) was demonstrated to be 0.717 (0.577–0.858). ROC: Receiver-operating characteristic.

**Figure 7.**
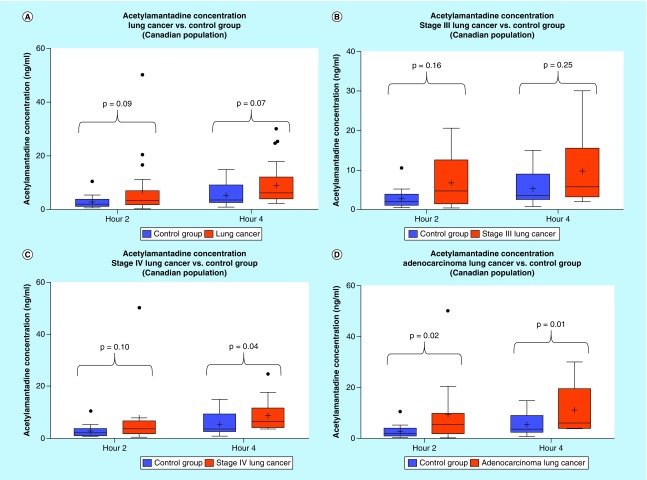
**Urinary acetylamantadine concentrations in patients with lung cancer from CancerCare Manitoba (Winnipeg site).** Acetylamantadine concentrations are shown for all types lung cancer and staging **(A)** and at Stage III **(B)**, Stage IV lung cancer **(C)** and in adenocarcinoma **(D)**. Urinary acetylamantadine concentrations were determined by LC–MS/MS technique at 2 and 4 h postamantadine ingestion. Values are mean ± SEM and are expressed as ng/ml. The p < 0.1 was considered as significantly different versus the healthy control group. LC–MS/MS: Liquid chromatography–mass spectrometry/mass spectrometry; SEM: Standard error mean.

For the comparisons of the data presented in Figure 1, Minitab version 15.1.0.0 was used. Summary statistics, for the data presented in Figures 2–7, were calculated for baseline patient characteristics along with the concentration and total amount of AA for all study cohorts. Mann–Whitney tests were performed to compare the control and cancer groups stratified by stage and type at hour 2, 4 and 6. In addition, receiver-operating characteristic (ROC) curves were developed to characterize the discriminatory ability of AA to differentiate control and cancer patients. A p-value less than 0.05 was considered significant. All statistical analysis was performed using SAS version 9.3. Figures 2–7 were developed using GraphPad Prism version 6.07 for Windows.

## Results

### Healthy control & cancer patient characteristics

The mean age (± SEM) of the healthy control group at the Winnipeg site (n = 20; 9 male, 11 female) was 38 ± 4 years, while the mean age (± SEM) of the healthy control group at the Bangladesh site (n = 29; 17 male, 12 female) was 45 ± 3 years. Selected demographics of the participants with cancer in this study are shown in Tables 1 and 2. Participants in the cancer group were older than the healthy control volunteers from both sites. Table 1 shows the different lung and breast cancer type as well as some staging information.

**Table 1.** **Demographic information on cancer patients recruited from the National Institute of Cancer Research & Hospital (Dhaka, Bangladesh site).**

**Lung cancer patients (n = 80)**	**n**
Age (year)	53 ± 1

Sex (female)	33

Cancer type	

Adenocarcinoma (n)	29

Nonsquamous (n)	10

Small-cell (n)	7

Squamous (n)	17

Mets (n)	2

Staging	

Early/Stage II (n)	6

Stage III (n)	7

Stage IV/advanced (n)	67

**Breast cancer patients (n = 39)**	

Age (year)	42 ± 2

Sex (female)	39

Breast cancer staging	

*DCC*/early	3

Stage II	2

Stage III	4

Stage IV/advanced	28

Age is presented as mean ± SEM. The cancer subtype information was not available for all lung cancer patients. Not all staging information was available for breast cancer patients.

Mets: Metastasized cancer.

**Table 2.** **Demographic information on cancer patients recruited from CancerCare Manitoba (Winnipeg site).**

**Lung cancer patients (n = 24)**	**n**
Age (year)	70 ± 2

Sex (female)	15

Lung cancer type	

Adenocarcinoma	14

Small-cell	4

Non-small cell	3

Squamous	2

NOS	1

Staging	

Stage I/II	1

Stage III	10

Stage IV	12

Age is presented as mean ± SEM. Staging information for one of the participants with lung cancer diagnosis was not available.

NOS: Not otherwise specified; SEM: Standard error mean.

### Urinary AA concentration in cancer patients

#### Pharmacokinetic analysis

In a previous study [18] conducted with healthy adult volunteers (n = 12), we investigated the influence of being in a fasted or fed state on urinary concentrations of AA over a 12 h period following ingestion of 200 mg (2 × 100 mg) amantadine. Figure 1A and B show that the T_max_ (the time after administration of a drug when the maximum plasma concentration is reached; when the rate of absorption equals the rate of elimination) was 2 and 4 h in the fasted and fed state, respectively. There were no significant differences in the other pharmacokinetic parameters for amantadine disposition. Amantadine relative bioavailability was not affected by being in either fed or fasted state (Figure 1C). Further analysis of the pharmacokinetic data revealed higher AA concentrations at 0–2 h after an oral dose of amantadine and the period of accelerated acetylation and rate of urinary excretion of AA (Figure 1D). Taken together, the 0–2 h period was identified as the optimal collection time for AA.

#### Urinary concentrations of AA in lung & breast cancer patients (Bangladesh site)

Analysis of the urine samples revealed that there was a higher concentration at 2 h of urinary AA in the lung cancer group as compared with healthy controls (Figure 2A). Although no difference was observed in the urinary AA concentrations in Stage II lung cancer patients, higher AA concentration was measured in patients with a Stage III and Stage IV diagnosis, but only at the 0–2 h postamantadine time point (Figure 3A). Interestingly, with respect to lung cancer subtypes, higher AA concentration was measured in patients diagnosed with squamous and adenocarcinoma lung cancer, again at 0–2 h postamantadine ingestion (Figures 3B & C). Although the mean value for AA concentrations at 2 h was higher in patients with nonsquamous and small cell lung cancer diagnosis, statistical significance was not reached (Figures 3A & D). The ROC curve for 2 h AA concentration demonstrated an area under the curve (AUC) of 0.689 (0.591–0.786, 95% CI [Figure 4]). Similarly, higher AA concentration was only measured at the 0–2 h time point in breast cancer patients as compared with healthy controls (Figure 5A); while a difference in the AA concentration was measured only in Stage IV diagnosed breast cancer patients. The ROC revealed an AUC of 0.717 (0.577–0858, 95% CI [Figure 6]). The two ROC curves developed and presented (Figures 4 & 6) were intended to characterize the ability of AA to differentiate between both lung cancer and breast cancer patients and healthy controls. The outcome was the presence of cancer, and the predictor of interest was 2 h AA for both models. Each point on the ROC curve represents different sensitivity/specificity trade-offs for both patient populations. For the lung cancer and breast cancer ROC curves, the likelihood ratio test χ^2^ values were 15.33 (p-value less than 0.001) and 8.20 (p-value of 0.004), respectively.

#### Urinary concentrations of AA in lung cancer patients (Winnipeg site)

Analysis of the lung cancer urinary AA concentration at the Winnipeg site revealed that a higher AA concentration was measured at both 2 and 4 h as compared with healthy adult controls (Figure 7A). Although the mean value for AA concentrations at 2 and 4 h was higher in patients with Stage III lung cancer diagnosis, statistical significance was not reached (Figure 7B). On the other hand, higher urinary AA concentration was observed with Stage IV diagnosed lung cancer patients at both 0–2 h and 2–4 h as compared with healthy adult controls (Figure 7C). Interestingly, with respect to lung cancer subtypes, higher AA concentration was seen in patients diagnosed with adenocarcinoma lung cancer at 0–2 h and 2–4 h postamantadine ingestion (Figure 7D).

## Discussion

We have recently reported that human cancer is associated with high urinary concentration of AA [15]. In the present study, we further validated the clinical utility of urinary AA concentration as a diagnostic marker for breast and lung cancer. Furthermore, unlike our previous work, urine was collected at 0–2, 2–4 and 4–6 h only at Bangladesh site after amantadine ingestion in order to determine the optimal time interval for detection of AA concentration as well as detection of the differences in AA concentration between breast and lung cancer patients and healthy adult controls. The results indicated that the 0–2 h postamantadine ingestion time interval for urine collection reflected the rapid onset of AA production and the optimal time point for urine collection that represented the higher amplification of the AA signal, due to increase SSAT-1 activity in cancer versus healthy control volunteers. The observations of the present study provide further evidence that amantadine can be used as a proxy to quantify indirectly the increased SSAT-1 acetylation activity in cancer patients as previously described [15] and further validates its use for the detection of breast and lung cancer. Of note, the data were particularly strong for adenocarcinoma lung cancer-diagnosed patients at both study sites. The differences observed in SSAT-1 activity were evident at higher stages of breast and lung cancer, but differences were not seen between healthy control and early stages of breast and lung cancer, which may be as a consequence of smaller sample size of patients with diagnosis of early-stage lung and breast cancer. In addition, our study enrolled newly diagnosed, untreated lung and breast cancer patients and was not prospectively recruiting patients at early-stage diagnosis, thus the recruitment was largely of patients with late stage cancer. In addition, due to smaller sample size it was not possible to determine if there was any correlation between SSAT-1 activity and the aggressiveness of either breast or lung cancer.

It should be pointed out that we did not observe any side effects with the single, one-time dose of amantadine [19,20] in either the healthy adults or cancer patients. In addition, we do not support the fact that amantadine (200 mg) as a single dose has any clinically relevant immunoregulatory activity. This assumption is based on the mechanism of antiviral activity of amantadine that blocks the entry of the virus into the cell, but is not known to inhibit any endogenous immune response to the viral infection [19]. Thus, there is no contraindication to the use of amantadine in cancer patients.

We have previously reported that amantadine is a specific substrate for SSAT-1 and can differentiate between acetylation by SSAT-1 and other *N*-acetyltransferases (NATs), since amantadine is not a substrate for acetylation by either NAT1 or NAT2 [12,13]. Thus, on the basis of our previous [15] and current findings amantadine acetylation may serve as a biomarker for cancer, since it occurs only as a result of SSAT-1 activity. It is pointed out that in our assay AA is not further metabolized and thus is the terminal end product of SSAT-1 activity. Accordingly, it is plausible that the AA signal can be presumed to be higher compared with other traditional cancer blood-based protein biomarkers, such as carcinoembryonic antigen, carbohydrate antigen 19-9 l and α-fetoprotein, alone [21–28]. Furthermore, these other biomarkers are considered to be unreliable, lacking sensitivity and specificity, which is required for early diagnosis of cancer. It should be noted that a patent has been filed that identifies several other potential SSAT-1 substrates, including rimantadine and tocainide; however, their specificity remains to be validated by further *in vitro* and *in vivo* studies in order to justify consideration of these substrates, other than amantadine, as suitable diagnostic tools for assessing upregulation of SSAT1.

It is important to note that approximately 25% of the healthy adult controls (11/40, Bangladesh site; 5/20, Winnipeg site) were deemed to be outliers with AA concentrations higher than expected. Accordingly, we have conducted a thorough examination of this group. At this point, a 6 and 9-month follow-up with the amantadine test has been conducted along with comprehensive clinical and hematological assessments. These findings are not in the scope of the present study, but are the focus of a subsequent paper. However, in this regard, it is interesting to note that we have previously observed regional/ethnic differences in AA concentration in healthy adults [18]; in order of magnitude of AA concentration: Bangladesh > Canada > China has been observed. However, the influence of genetics, environmental and lifestyle practices on baseline AA concentration in healthy adults remains to be fully understood and thus warrants further investigation.

While current standards for determining and verifying the presence of cancer involve computed tomography (CT), MRI and ultrasound in conjunction with other molecular and protein biomarkers, they are expensive, often inaccessible, involve exposure to ionizing radiation and have an unacceptable level of false positives. In addition, biopsies for molecular and biomarker diagnosis are invasive and uncomfortable. Clearly, CT screening is capable of identifying early lung cancers. The issue of overdiagnosis is a limiting factor, generating anxiety for patients and adding expense for the healthcare system [6]. The race is on to develop a biological tool that would be capable of improving the specificity of CT screening for lung cancer. Contenders include plasma biomarkers such as circulating cell-free DNA (cfDNA). It has been suggested that this type of assay is capable of differentiating between benign and malignant CT-identified lung lesions [29,30]. With this type of assay, positivity has been shown to correlate with very aggressive tumors, increasing tumor burden and specificity has been an issue [31]. An ideal test should be capable of identifying the smallest malignant lung lesions. Analysis of epigenetic markers is another promising approach. Options here include analysis of micro-RNA [32] and protein/enzymatic assays, such as is described herein. Validation and incorporation of one of these biomarker assays is urgently needed to realize the full potential of screening for lung cancer. Our findings provide further evidence on the use of amantadine acetylation as a simple screening test for detecting the presence of breast and lung cancer. It is also possible that the test could serve as a surveillance test in populations at high risk for developing cancer and monitoring patients after cancer intervention to detect potential proliferation of new cancer cells. In addition, the quantification of SSAT-1 activity, especially in patients with lung cancer could serve as a prognostic tool for determining stage of the disease as well as a diagnostic tool for testing for the presence of lung and possibly breast cancer. In view of the projected global increase in death due to lung cancer by 2040 [3], our proposed test may be increasingly important with time in helping to detect the disease perhaps at an earlier stage when it is more likely to be curable and therefore needs to be further investigated.

On the other hand, the polyamine pathway has been suggested to be an attractive target for the development of novel anticancer therapies [33]. Indeed, polyamines such as squalamine, putrescine and spermidine have been used as inhibitors of SSAT-1 and have been shown to exhibit *in vitro* anticancer activities [34–36], and to inhibit human tumor xenograft growth [37]. Although a number of other inhibitors targeting individual enzymes in the polyamine pathway exist [38,39], they have had limited success [38]. Therefore, the regulation of SSAT-1 expression and its activity have been explored as a novel and highly effective approach in treating specific cancers [40,41]. Inhibition of SSAT1 would be expected to result in a series of cascading effects in cancer tissues including accumulation of intracellular polyamines and increase in polyamine-mediated cytotoxicity, a possibility that warrants further investigation.

## Conclusion

The amantadine acetylation test is a less expensive, noninvasive and simple to use tool with high clinical applicability and significance that can detect the presence of breast and lung cancer, while on the basis of our findings SSAT-1 may also serve as an important and novel therapeutic target for at least breast and lung cancer.

## Future perspective

Our earlier and present findings have provided evidence for the use of amantadine as a simple screening test for the detection of cancer as well as potentially a surveillance test in populations at high risk for developing cancer. It is also very possible that the test could be used for monitoring patients after surgical or chemo/radiation therapy to detect not only the response of the tumor to the intervention, but it could also have the possibility to detect the proliferation of new cancer cells. This is currently under investigation. It is also pointed out that we are in late development stage of including specific metabolites within the polyamine pathway metabolomic fingerprinting panel that will further the robustness of the assay performance. While the ROC curves presented in this study are for the single metabolite (AA), it is anticipated that inclusion of two or more metabolites within the polyamine pathway will increase AUC values further.

**Summary points**

- Lung cancer is the leading cause of cancer death among both men and women.
- Currently lung cancer ranks 13^th^ as a leading cause of death globally and by 2040 is projected to rise to the 9^th^ leading cause of death.
- Breast cancer is the most common cancer in women worldwide.
- The utility of amantadine as an agent to demonstrate increased SSAT-1 activity linked to these cancers was performed.
- Higher urinary concentration level of AA is detectable in patients with different subtypes of lung cancer and breast cancer, which may also be related to staging.
- The detection of AA has the potential as a novel, simple-to-use test for the presence of lung and breast cancer.
